# Foods and beverages associated with smoking craving in heated tobacco product and cigarette smokers: A cross-sectional study

**DOI:** 10.18332/tid/175623

**Published:** 2024-01-05

**Authors:** Kiho Miyoshi, Yuki Kimura, Misaki Nakahata, Takashi Miyawaki

**Affiliations:** 1 Department of Living Environment, Graduate School of Home Economics, Kyoto Women’s University, Kyoto, Japan; 2 Department of Food and Nutrition, Graduate School of Home Economics, Kyoto Women’s University, Kyoto, Japan; 3 Department of Pharmacoepidemiology, Graduate School of Medicine, Kyoto University, Kyoto, Japan

**Keywords:** smoking, heated tobacco product, food, taste, beverage

## Abstract

**INTRODUCTION:**

Certain foods and beverages are associated with smoking craving. However, only few studies have explored the relationship between food or beverage-related and taste-associated smoking craving. In this study, we aimed to identify the types of foods related to smoking craving in Japanese individuals who smoke cigarettes or heated tobacco products (HTPs).

**METHODS:**

A total of 657 individuals (HTP and cigarette smokers and never smokers) participated in this cross-sectional study. Participants were asked what foods/beverages, tastes, seasonings, cooking methods, and cuisine categories, made them want to smoke and what foods they consumed.

**RESULTS:**

Alcoholic beverages such as beer, coffee, and fat-rich foods were associated with a higher likelihood of smoking craving. Fruits, dairy products such as milk, and sweet and sour tastes, were associated with a lower likelihood of smoking craving. The daily intake of fruit and dairy products was significantly lower in cigarette and HTP smokers than in non-smokers (median fruit intake: non-smokers, 46.4 g/1000 kcal/day; cigarette smokers, 22.2 g/1000 kcal/day; HTP smokers, 31.4 g/1000 kcal/day; p<0.001; median dairy product intake: non-smokers, 76.3 g/day; cigarette smokers, 48.2 g/day; HTP smokers, 57.6 g/day; p<0.001) as assessed using a food frequency questionnaire (BDHQ).

**CONCLUSIONS:**

Specific foods and beverages such as alcohol, fruits, and dairy products are related to smoking craving, and their intake differs according to smoking status.

## INTRODUCTION

Cigarette smoking causes respiratory diseases, such as lung cancer^1^, and cardiovascular diseases^2^. Therefore, quitting smoking is important to ensure good health. However, tobacco leaves contain nicotine: one of the alkaloids that combine at nicotinic acetylcholine receptors (nAChRs)^3^ and induce secretion of dopamine, a neurotransmitter in the brain that induces pleasure, thus causing addiction^4^. Chronic smoking leads to nicotine addiction, comparable in strength to heroin, cocaine, or alcohol addiction, and is associated with challenges in cessation similar to those for these substances^5^. Recently, heated tobacco products (HTPs) have been developed in Japan that do not involve burning tobacco leaves but instead use a specialized instrument for heating tobacco, with resultant aerosols being inhaled by smokers. Although some HTP health hazards have been identified, such as vascular endothelial function impaired by aerosol from a single stick^6^, other adverse health effects cannot be ruled out.

Smoking craving can occur in various situations. Notably, alcohol or coffee consumption targets nAChRs and induces smoking craving^7,8^. Therefore, smoking behavior and craving are closely associated with the consumption of certain foods and beverages. However, only few studies have investigated foods and beverages in relation to smoking craving^9^, and effective nutritional therapy to control smoking craving has not been established. Importantly, previous studies only addressed cigarettes, not HTPs. Furthermore, no study has investigated the taste, seasoning, cooking method, and cuisine category associated with smoking craving.

In this study, we aimed to facilitate smoking cessation guidance from a nutritional perspective. Therefore, we investigated the following: 1) the types of food and beverages associated with a higher or lower likelihood of smoking craving, and 2) the differences in food and beverage choices that were associated with a higher or lower likelihood of smoking craving between cigarette–HTP smokers and never smokers.

## METHODS

### Participants

**Figure 1:**
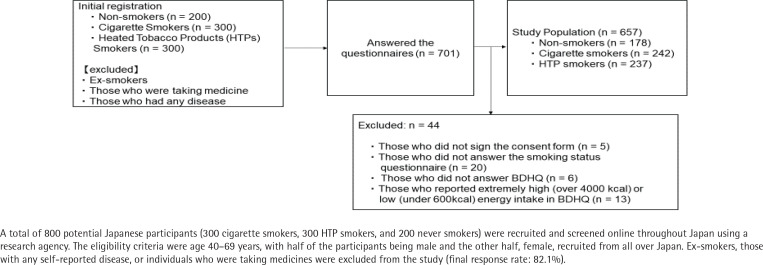
Study flow diagram of the registration of participants in Japan, 2022 (N=657)

A total of 800 potential Japanese participants (300 cigarette smokers, 300 HTP smokers, and 200 never smokers) were recruited and screened online throughout Japan using a research agency (Asmarq Corp., Tokyo, Japan). The eligibility criteria were age 40–69 years, with half of the participants being male and the other half, female, recruited from all over Japan. Ex-smokers, those with any self-reported disease, or individuals who were taking medicines were excluded from the study. Questionnaires were sent by mail to eligible participants, and 701 of them responded (response rate: 87.6%). Respondents who did not sign the consent form (n=5), did not answer the question about their smoking status (n=20) or habitual eating questionnaire (Brief Self-Administered Diet History Questionnaire [BDHQ]^10,11^; n=6), and reported extremely high (>4000 kcal) or low (<600 kcal) energy intake in the BDHQ (n=13) were excluded. The final study population comprised 178 never smokers, 242 cigarette smokers, and 237 HTP smokers (n=657) (final response rate: 82.1%) (Figure 1). We set up a catchment pool at our university, and the participants mailed their completed questionnaires to our university. Written informed consent was obtained from all participants. This study was approved by the Research Ethics Committee of the of Kyoto Women’s University and was performed in accordance with the guidelines of the Declaration of Helsinki.

### Questionnaires

The participants were asked to complete three questionnaires. First, smokers were asked which foods or beverages made them want to smoke. The questionnaire was developed to investigate food preferences related to smoking craving by referring to previous studies^9^ and based on the food item categories in the BDHQ. In the questionnaire, we investigated foods and beverages associated with smoking craving. For the question: ‘Do you feel like smoking when you eat/drink the following items?’, the participants were given the options ‘Yes’, ‘Neutral’, ‘No’, and ‘I don’t know/I don’t eat (owing to religious, cultural, allergy, or other reasons)/I don’t like’. Those who answered ‘I don’t eat’ in each category were excluded. In addition to asking about foods and beverages, we asked about taste, seasoning, cooking methods, and cuisine categories associated with smoking craving. Sweet, salty, sour, bitter, umami, oily, spicy, hot, and astringent items were included in the taste options. The seasoning options were sugar, salt, vinegar, soy sauce, miso, mayonnaise, and sauces. For cooking methods, fried, stir-fried, grilled, simmered, steamed, dressed, and boiled were included as options. For the cuisine category, Japanese, Chinese, Western, and Korean options were provided. Second, habitual dietary/food intake data were obtained using the BDHQ for never smokers, cigarette smokers, and HTP smokers. The BDHQ is a four-page structured self-administered questionnaire that estimates the dietary intake of 58 very common food and beverage items in Japan^10^. The BDHQ assesses dietary habits during the preceding month, and consists of the following five sections: 1) intake frequency of 46 food and non-alcoholic beverage items; 2) daily intake of rice, including type of rice (refined or unrefined, etc.), and miso soup; 3) frequency of alcoholic beverage drinking and amount per drink for five alcoholic beverages; 4) usual cooking methods; and 5) general dietary behavior^10^. The food intake results from the BDHQ are shown in g/day and the beverage intake is shown in mL/day. The BDHQ has been validated previously^10,11^. Third, the participant characteristics obtained included sex, age, body weight, height, smoking status, and body mass index (BMI, kg/m^2^).

### Statistical analyses

The sample size in this study was calculated based on the formula for a cross-sectional study (α=0.05). Data were analyzed using IBM SPSS version 26 (IBM Corp., Armonk, NY, USA). Pearson’s chi-squared test was used to compare the proportion of respondents who answered ‘Yes’ to the question, ‘Do you feel like smoking when you eat/drink the following items?’, by cigarette type (cigarettes or HTPs).

Data were tested using the Shapiro-Wilk normality test, and a normal distribution was not found. Therefore, data are presented as the median and interquartile range (IQR). The Kruskal-Wallis test and Bonferroni correction were used to assess significant intergroup differences in the characteristics of participants and dietary food intakes. All tests were two-tailed. Statistical significance was set at p<0.05.

## RESULTS

### Participant characteristics

Approximately half of the study cohort comprised female participants (males: 322; females: 335). The median ages of the men and women were 53 and 52 years, respectively. No significant intergroup differences in height, body weight, or BMI, based on the smoking status and type of cigarettes were observed (Table 1).

**Table 1:** Participant characteristics, Japan, 2022 (N=657)

*Characteristics*	*Total n*	*All (N=657) Median (IQR)*	*Never smokers (N=178) Median (IQR)*	*Cigarette smokers (N=242) Median (IQR)*	*HTP smokers (N=237) Median (IQR)*	*p*
**Age** (years)						
Males	322	53.0 (48.0–62.0)	53.0 (47.5–62.0)	54.0 (48.0–61.0)	53.0 (47.8–62.0)	0.912
Females	335	52.0 (46.0–60.0)	54.0 (47.0–61.0)	53.0 (46.0–61.0)	51.0 (46.0–57.0)	0.155
**Height** (cm)						
Males	89	170.0 (168.0–174.1)	169.9 (167.0–174.5)	171.0 (168.0–174.7)	171.0 (168.0–174.0)	0.472
Females	89	158.0 (155.0–161.5)	157.0 (153.0–161.8)	158.7 (155.0–161.0)	158.0 (156.0–162.0)	0.188
**Weight** (kg)						
Males	115	66.0 (60.4–73.1)	67.0 (60.0–74.3)	65.0 (60.0–72.8)	67.0 (62.9–73.6)	0.427
Females	127	51.0 (46.0–56.0)	51.0 (46.0–56.7)	50.4 (45.0–55.5)	50.3 (46.8–56.0)	0.813
**BMI** (kg/m^2^)						
Males	118	22.8 (21.1–24.8)	23.0 (20.8–25.4)	22.3 (20.8–24.2)	23.1 (21.4–25.2)	0.119
Females	119	20.2 (18.7–21.9)	20.6 (18.9–22.7)	20.1 (18.4–22.0)	20.0 (18.8–21.6)	0.336

BMI: body mass index. HTP: heated tobacco product. IQR: interquartile range. Data were tested using the Shapiro-Wilk normality test, and a normal distribution was not found. Therefore, data are presented as median (IQR). The Kruskal-Wallis test and Bonferroni correction were used to assess significant intergroup differences in characteristics. Statistical significance was set at p<0.05.

### Foods and beverages that altered smoking craving

*Foods and beverages/tastes/seasonings/cooking methods/cuisine categories associated with higher likelihood of smoking craving*

**Figure 2:**
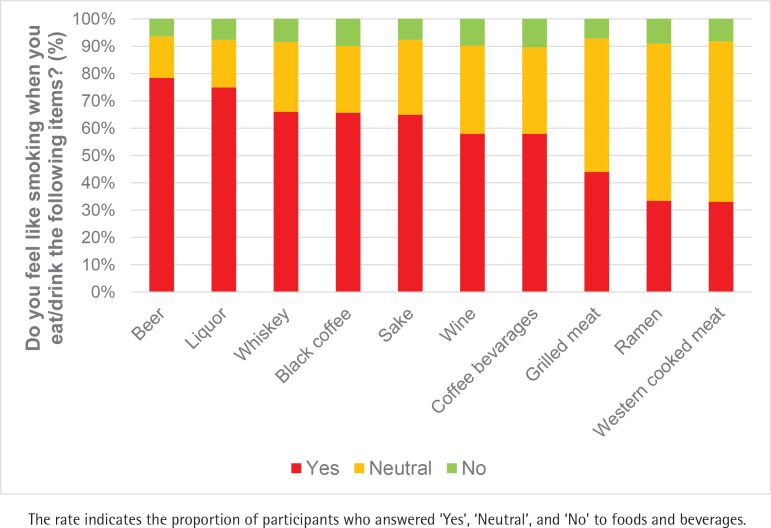
Foods and beverages that were associated with a higher likelihood of smoking craving in Japan, 2022 (cigarette smokers and HTP smokers, N=479)

Figure 2 shows the top 10 food and beverage items that were associated with a higher likelihood of smoking craving. Seventy-eight percent of smokers answered ‘Yes’ to beer as the leading beverage that induced smoking craving. Moreover, liquor (74.9%), whiskey (66.0%), black coffee (65.6%), sake (64.9%), wine (58.0%), and coffee beverages (58.0%) were selected as beverage items that were associated with a higher likelihood of smoking craving. The major popular foods that were associated with a higher likelihood of smoking craving were grilled meat (44%), ramen noodles (33.3%), and Western-style cooked meats (33.0%).

**Figure 3:**
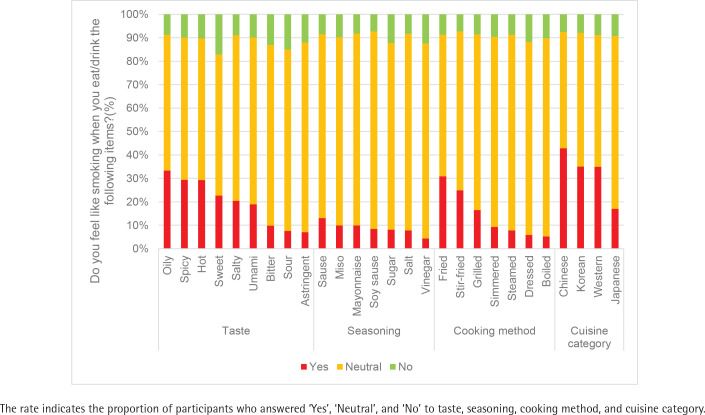
Taste, seasoning, cooking method, and cuisine categories associated with a higher likelihood of smoking craving in Japan, 2022 (cigarette smokers and HTP smokers, N=479)

Figure 3 displays the leading taste, seasoning, cooking method, and cuisine category that heightened smoking craving, as follows: oily (33.3%), sauce (13.0%), fried (30.9%), and Chinese (42.9%), respectively.

*Foods and beverages/tastes/seasonings/cooking methods/cuisine categories associated with lower likelihood of smoking craving*

**Figure 4:**
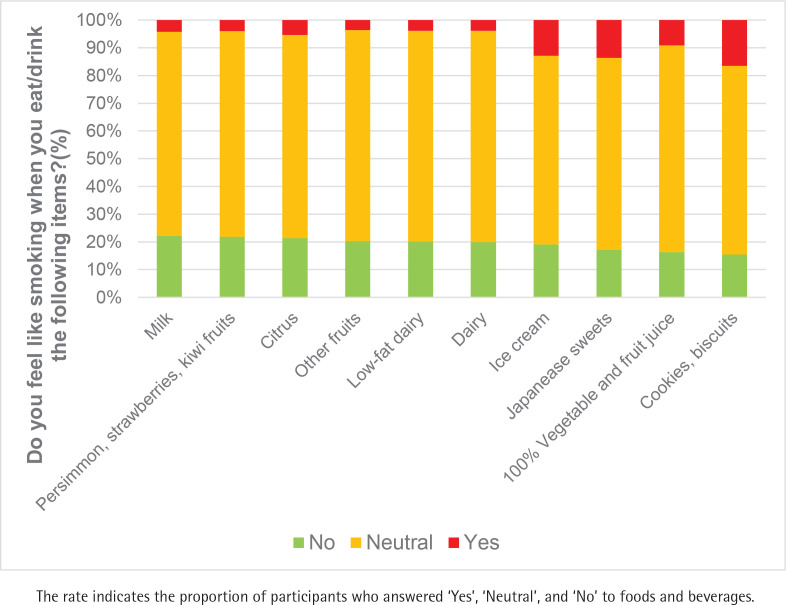
Foods and beverages that were associated with a lower likelihood of smoking craving in Japan, 2022 (cigarette smokers and HTP smokers, N=479)

Figure 4 shows foods and beverages that were associated with a lower likelihood of smoking craving. The major items were milk (22.2%) followed by persimmons, strawberries, and kiwifruit (21.8%), citrus fruits (21.3%), other fruits (20.4%), low-fat dairy products (20.3%), regular and high-fat dairy products (19.9%), ice cream (19.1%), Japanese sweets (17.1%), and 100% fruit and vegetable juice (17.1%). Dairy products and fruits were the top choices; however, the percentage of respondents who selected them was low (around 20%).

**Figure 5:**
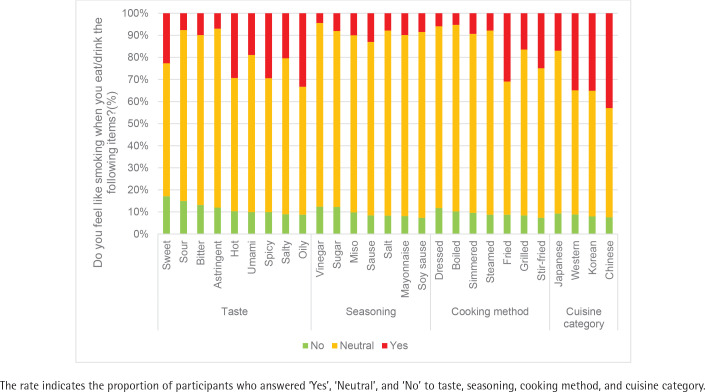
Taste, seasoning, cooking method, and cuisine categories associated with a lower likelihood of smoking craving in Japan, 2022 (cigarette smokers and HTP smokers, N=479)

The major tastes, seasonings, cooking method, and cuisine category that were associated with a lower likelihood of smoking craving were sweet (17.1%) and sour (15.0%), vinegar (12.4%) and sugar (12.2%), dressed (11.8%), and Japanese (9.2%), respectively. However, the percentage of participants who selected them was low (Figure 5).

### Intake of items that altered smoking craving

The consumption of key items, such as alcoholic beverages, was associated with a higher likelihood of smoking craving, while the consumption of dairy products and fruits was associated with a lower likelihood of smoking craving, varying by the type of tobacco product used (Table 2). The intake of alcoholic beverages was significantly higher in cigarette and HTP smokers than in never smokers (median intake: never smokers, 10.3 mL/1000 kcal/day; cigarette smokers, 76.6 mL/1000 kcal/day; and HTP smokers, 39.4 mL/1000 kcal/day; p<0.001).

**Table 2:** Alcoholic beverages, milk, and fruits intake by type of smoking in Japan, 2022 (N=657)

*Consumption*	*All (N=657) Median (IQR)*	*Never smokers (N=178) Median (IQR)*	*Cigarette smokers (N=242) Median (IQR)*	*HTP smokers (N=237) Median (IQR)*	*p*
**Alcoholic beverages** (mL/1000 kcal/day)	35.7 (0.0–180.3)	10.3 (0.0–89.1)	76.6 (0.0–209.6)	39.4 (0.0–193.9)	<0.001
**Dairy products** (g/1000 kcal/day)	65.2 (15.0–109.4)	76.3 (39.5–120.0)	48.2 (7.8–104.1)	57.6 (14.7–108.8)	<0.001
**Fruits** (g/1000 kcal/day)	31.8 (9.7–71.7)	46.4 (18.1–81.7)	22.2 (6.7–63.9)	31.4 (8.7–70.9)	<0.001

IQR: interquartile range. Data were tested using the Shapiro-Wilk normality test, and a normal distribution was not found. Therefore, data are presented as median (IQR). The Kruskal-Wallis test and Bonferroni correction were used to assess significant intergroup differences in food intakes. Statistical significance was set at p<0.05.

The intake of dairy products and fruits was significantly lower in cigarette and HTP smokers than in never smokers (median dairy product intake: never smokers, 76.3 g/day; cigarette smokers, 48.2 g/day; HTP smokers, 57.6 g/day; p<0.001; median fruit intake: never smokers, 46.4 g/1000 kcal/day; cigarette smokers, 22.2 g/1000 kcal/day; and HTP smokers, 31.4 g/1000 kcal/day; p<0.001).

### Comparison of items associated with smoking craving by tobacco type

The proportion of respondents who answered ‘Yes’ to the question, ‘Do you feel like smoking when you eat/drink the following items?’ was compared by cigarette type (cigarette or HTP). The use of ramen, spaghetti, water, grilled meat, western meat, and stir-fried meat was significantly higher in HTP smokers than in cigarette smokers (cigarette smokers vs HTP smokers: ramen: 28.5% vs 38.4%, p=0.025; spaghetti: 16.1% vs 23.7%, p=0.042; water: 1.7% vs 5.5%, p=0.033; grilled meat: 39.3% vs 48.9%, p=0.038; Western meat: 28.1% vs 38.1%, p=0.023; stir-fried meat: 22% vs 30.8%, p=0.032). For seasonings, sauces with tastes described as bitter, hot, spicy, and oily demonstrated significant intergroup differences (bitter: 6.5% vs 13.3%, p=0.019; hot: 24.8% vs 33.8%, p=0.038; spicy: 23.3% vs 35.6%, p=0.004; oily: 28.5% vs 38.4%, p=0.025). In terms of cooking methods, stir-fried and fried dishes also showed notable differences (stir-fried: 20.7% vs 29.3%, p=0.031; fried: 26.7% vs 35.3%, p=0.047). Lastly, in the cuisine category, both Western and Korean dishes exhibited significant disparities (Western: 28.6% vs 41.6%, p=0.003; Korean: 29.8% vs 40.4%, p=0.024) in the rates of respondents answering ‘Yes’. These items were associated with a significantly higher likelihood of smoking craving in HTP smokers compared to those of cigarette smokers after eating. No items received a higher ‘Yes’ response rate from cigarette smokers to the question, ‘Do you feel like smoking when you have the following items?’ than did the HTP smokers.

## DISCUSSION

This is the first study to investigate food and beverage items/tastes/cooking methods/seasonings/cuisine categories associated with smoking craving and examine the food item intake associated with smoking craving of HTP smokers in all regions of Japan. This study revealed that alcoholic beverages, such as beer, coffee, grilled meat, ramen noodles, and Western-style cooked meat, were associated with a higher likelihood of smoking craving. Sauces, concerning seasoning, fried foods as a cooking method, oily flavors for taste, and Chinese for cuisine category were associated with a higher likelihood of smoking craving. In contrast, fruits, milk and other dairy products, vinegar for seasoning, dressed foods as the cooking method, sweet and sour flavors for taste, and the Japanese cuisine category were associated with a lower likelihood of smoking craving. The intake of fruits and milk was significantly lower in cigarette and HTP smokers than in never smokers.

Nicotine profoundly impacts brain neurochemistry by activating nAchRs, which are widely distributed throughout the brain, leading to dopamine release in both the brain and the nucleus accumbens. This effect is similar to that produced by other abused substances (e.g. amphetamines and cocaine) and is a critical feature of the addiction mechanism^12^. Nicotine binds to nAchRs and decreases appetite and food intake through activating pro-opiomelanocortin neurons^13^; therefore, smoking behavior, food intake, and appetite are closely connected.

In this study, alcohol and coffee were the top two items that were associated with a higher likelihood of smoking craving. A previous study reported that alcohol and caffeinated beverages enhanced cigarette taste^9^. Specifically, ethanol has been suggested as an activator of cholinergic afferents, causing the release of acetylcholine in the ventral tegmental area, leading to nAChR stimulation, followed by excitation of the mesocorticolimbic dopamine system^14^. Similarly, caffeinated beverages, such as coffee, combine with the adenosine receptor as an antagonist and inhibit adenosine as well as the release of dopamine. Thus, caffeine affects gamma-aminobutyric acid, glutamate, and dopamine levels^15^. A previous study suggested that addictive substances such as coffee (caffeine), alcohol, and smoking interact^8,16,17^, which is consistent with the faster metabolism of caffeine by smokers, and that n-MP, a substance in coffee beans, affects nicotinic acetylcholine receptors^8^. These biochemical mechanisms may explain why alcohol and coffee are associated with a higher likelihood of smoking craving.

The present study revealed that foods high in fats and oils (based on the results of taste, Chinese foods, and fried foods) may be associated with a higher likelihood of smoking craving, whereas fruits and dairy products may be associated with a lower likelihood of smoking craving. The consumption of fruit and dairy products was significantly lower in cigarette and HTP smokers than in never smokers. Previous preclinical and clinical studies have demonstrated that stress induction increases the proclivity of high-fat and high-sugar foods^18,19^. Smokers often feel stressed due to nicotine withdrawal, which may induce a preference for oils or fats. Furthermore, smokers have a reduced sense of taste^20^. Foods with fat that are oil-in-water soluble are known to increase sensitivity to sweet, salty, sour, and umami tastes and conversely decrease bitterness^21^, and linoleic and oleic acids may alter the licking responses to sweet, salt, sour, and bitter tastes^22^, which may explain why smokers consume foods with high oil and fat content to perceive taste better and improve the perception of declining taste, in addition to reducing the bitterness of cigarettes. A previous study has also shown that foods high in fats and oils, such as meat, make cigarettes taste better^9^.

Fruits and dairy products, vinegar and sugar, sweet and sour tastes, dressed foods, and Japanese meals were items that reduced smoking craving. The common features of these items are that they are sour or sweet, refreshing, and low in fat. The proportion of free nicotine for easy absorption is higher when smoke is alkaline, and nicotine absorption depends on salivary pH^23,24^. Fruits, dairy products, and vinegar contain organic acids such as citric acid, malic acid, lactic acid, butyric acid, and acetic acid, may decrease nicotine absorption and explain why smokers avoid or consume few of these items.

In this study, the percentage of items that were associated with a higher likelihood of smoking craving was high (almost 80%), although that of items that were associated with a lower likelihood of smoking craving was low (approximately 20%). Dopamine is secreted when eating meals (brain reward circuit). Previous research^25^ using functional magnetic resonance imaging has shown that while activation of the reward circuit (ventral striatum) appears in response to tobacco-related rewards in nicotine dependence, responses to rewards other than tobacco (e.g. food and money) are reduced. Therefore, smokers are less satisfied with their meals (Paradise Lost theory)^26,27^. This may be the reason why smokers report that smoking craving occurs after meals (to secrete dopamine and feel the satisfaction that was not obtained from the meal).

Among foods that were associated with a higher likelihood of smoking craving, significant differences were found in the following categories: ramen noodles, grilled meat, sauces, hot, spicy, and oily flavors, Western foods, and Korean foods according to tobacco type. All the items for which significant differences were observed had a higher percentage for the HTP smokers than they did for the cigarette smokers. The items for which significant differences were found may be characterized by strong and oily flavors. There are two possible explanations for this discrepancy. First, a previous report showed that the nicotine levels delivered to the aerosol by HTPs were 70–80% of those from cigarettes^28^, and only 72% of HTP smokers were satisfied by smoking^29^, suggesting that they may be trying to compensate for the lack of satisfaction from smoking with satisfaction from food with a strong flavor. Second, HTPs contain more propylene glycol and glycerol than cigarettes do^28^, taste sweet, and may affect the taste perception of HTP smokers. Further studies are required to clarify smokers’ tastes and food preferences using HTPs.

### Limitations

This study had several limitations. The participants were Japanese individuals, and the results may not be generalizable to individuals with different eating habits. Moreover, the food categories used in this study are only common in Japan, and food item combinations have not been considered. The relationship between food intake/preferences and smoking status, such as smoking history, cigarettes per day, brand of cigarettes, and addiction levels (strengthening of nicotine withdrawal symptoms and stress) was not investigated. In addition, sex differences were not explored. This study had a cross-sectional design. We did not investigate causal relationships, and residual confounding was not addressed by regression models. Furthermore, the age range of participants was 40–69 years, which is quite broad and specific, not reflecting the general smoking population. The reliability and internal consistency of the questionnaire were not checked. However, BDHQ, on which the food categories were based, has been validated previously. Although smoking craving may be related to nicotine levels in the body or other factors such as environmental situations, the questionnaire used in this study was a subjective assessment and did not provide an objective evaluation.

Despite these limitations, this study identified the food and beverage products, tastes, seasonings, cooking methods, and cuisine categories that were associated with smoking craving. In addition, food preferences from food intake related to smoking craving differed between cigarette smokers and HTP smokers, suggesting that, in addition to smoking status, it is important to consider the smoking method, food preferences, and diet when providing nutritional and smoking cessation guidance. Future studies such as biochemical, pathophysiological, and nutritional perspectives are needed to assess the relationship between the mentioned foods and smoking craving in both smokers and individuals in the smoking cessation process.

## CONCLUSIONS

This study revealed that specific foods and beverages, taste, cooking method, seasoning, and cuisine category were associated with smoking craving and that their intake was correlated. Moreover, the results differed between cigarette and HTP smokers. Further studies are needed to explore the physiological mechanisms by which specific foods are associated with smoking craving.

## Data Availability

The data supporting this research cannot be made available for privacy or other reasons.
